# Recognizing Information Feature Variation: Message Importance Transfer Measure and Its Applications in Big Data

**DOI:** 10.3390/e20060401

**Published:** 2018-05-24

**Authors:** Rui She, Shanyun Liu, Pingyi Fan

**Affiliations:** Department of Electronic Engineering, Tsinghua University, Beijing 30332, China

**Keywords:** information transfer measure, small probability events, big data analysis and processing, mobile edge computing (MEC), queue theory

## Abstract

Information transfer that characterizes the information feature variation can have a crucial impact on big data analytics and processing. Actually, the measure for information transfer can reflect the system change from the statistics by using the variable distributions, similar to Kullback-Leibler (KL) divergence and Renyi divergence. Furthermore, to some degree, small probability events may carry the most important part of the total message in an information transfer of big data. Therefore, it is significant to propose an information transfer measure with respect to the message importance from the viewpoint of small probability events. In this paper, we present the message importance transfer measure (MITM) and analyze its performance and applications in three aspects. First, we discuss the robustness of MITM by using it to measuring information distance. Then, we present a message importance transfer capacity by resorting to the MITM and give an upper bound for the information transfer process with disturbance. Finally, we apply the MITM to discuss the queue length selection, which is the fundamental problem of caching operation on mobile edge computing.

## 1. Introduction

In recent years, due to the exploding amount of data, computing complexity for data processing is growing rapidly. In particular, cloud data center traffic will jump up to one order of magnitude by 2020 [1,2]. To some degree, the reason for this phenomenon seems to be that more and more mobile devices such as smartphones, tablets or mobile Internet of things (IoT) devices are utilized and the growing services of clouds are provided. In this context, it is necessary to dig out the valuable information from the collected data. On one hand, computation technologies including cloud computing and mobile edge computing (MEC) are needed for big data processing. On the other hand, it is essential to develop more efficient technologies for big data analysis and mining, such as distributed parallel computing, machine learning, deep learning, and neural networks, etc. [3,4,5,6].

As for data mining, the small probability events usually attract much more attention than the large probability ones [7,8,9,10]. In other words, there exits higher practical value in the rarity of small probability events. For example, in the anti-terrorist scenario, we just focus on a few illegal and dangerous people [11]. Moreover, as for the synthetic identification (ID) detection, only a small number of artificial identities for financial frauds should be paid more attention to [12]. In fact, it is challenging and significant to measure and mine small probability events.

According to rate-distortion theory, it is rational for us to regard small probability events detection as a clustering problem [13,14]. By using popular clustering principles (e.g., minimum within-cluster distance, maximum inter-cluster distance, and minimum compressing distortion), some efficient clustering approaches were proposed to detect small probability events. Specifically, a graph-based rare category detection and time-flexible rare category detection were presented based on the global similarity matrix and time-evolving of graphs, respectively [15,16]. Actually, these algorithms were proposed by resorting to traditional information measures and theory, which are considered from the viewpoint of typical events, which are the large probability events.

### 1.1. Information Transfer Measures Based on Message Importance

#### 1.1.1. Review of Message Importance Measure

In information theory, there are two fundamental measures, Shannon entropy and Renyi entropy, which have a vital impact on wireless communication, estimation theory, signal processing and pattern recognition etc. Nevertheless, they are not applicable to mining small probability events hidden in big data.

To do this, a new information measure named message importance measure (MIM) is proposed from the perspective of big data [17]. To simplify the form of MIM, we shall introduce the definition of MIM as follows.

**Definition** **1.** *For a continuous probability distribution f(x) with respect to the variable X in a given interval $S_{x}$, the differential message importance measure (DMIM) focusing on the small probability events is defined as*

(1) $$\begin{array}{cc} L(f(x)) & =\int_{S_{x}}f\left(x\right)e^{-f(x)}dx,\;\;x\in S_{x}. \end{array}$$

*Furthermore, for the discrete probability P=$\{p\left(x_{1}\right)$, $p(x_{2})$, …,$p(x_{n})\}$, the relative message importance measure (RMIM) is given by*

(2) $$\begin{array}{cc} L(P) & =\sum_{x_{i}}p\left(x_{i}\right)e^{-p(x_{i})}. \end{array}$$

By resorting to the exponential form, the MIM can amplify small probability elements much more than Shannon entropy and Renyi entropy, which include the logarithm operator or polynomial operator respectively. Actually, this highlights the significance of small probability events in information measure and theory. In addition, a series of postulates are investigated to characterize Shannon entropy and Renyi entropy. Particularly, Fadeev’s postulates are well-known to describe the information measures, which consist of four postulates [18]. In this case, in terms of two independent random distributions *P* and *Q*, there exists a weaker postulate for Renyi entropy than that for Shannon entropy, as follows

(3) H(PQ)=H(P)+H(Q),

where the function H(·) denotes a kind of information measure. Similarly, there exists a weaker postulate for the MIM than that for Renyi entropy, namely

(4) H(PQ)≤H(P)+H(Q).

Consequently, from the viewpoint of generalized Fadeev’s postulates, we can regard the MIM as a reasonable information measure similar to Shannon entropy and Renyi entropy.

#### 1.1.2. Message Importance Transfer Measure

As for an information transfer process, we construct such a model that the original probability distribution *P* and the final one *Q* in the transfer process satisfies the Lipschitz condition as follows,

(5) $$\left|H\left(P\right)-H\left(Q\right)\right|\leq {\lambda ∥P-Q∥}_{1},$$

where H(·) is the corresponding information measure function; λ>0 is the Lipschitz constant; ${∥\cdot ∥}_{1}$ denotes the $l_{1}$-norm measure.

Here, we shall analyze and measure the information transfer process mentioned in Equation (5) from the perspective of the message importance. In fact, it is a significant problem for us to measure the message importance variation in big data analytics. According to Definition 1, it is available to regard the DMIM or RMIM as an element to measure the message importance distance which can be also used in the discussion of information transfer processes. Then, an information transfer measure focusing on the message importance are proposed as follows.

**Definition** **2.** *For two probability distributions g(x) and f(x) with respect to the variable X in a given interval $S_{x}$, the message importance transfer measure (MITM) is defined as*

(6) $$\begin{array}{c} D_{I}\left(g\left(x\right)||f\left(x\right)\right)\\ =L(g(x))-L(f(x))\\ =\int_{S_{x}}\left(g\left(x\right)e^{-g(x)}-f\left(x\right)e^{-f(x)}\right)dx,x\in S_{x}. \end{array}$$

*Furthermore, in terms of the two discrete probability $Q=\{q\left(x_{1}\right),q\left(x_{2}\right),\ldots ,q\left(x_{n}\right)\}$ and $P=\{p\left(x_{1}\right),p\left(x_{2}\right),\ldots ,$$p(x_{n})\}$, the MITM can be written as*

(7) $$\begin{array}{c} D_{I}\left(Q||P\right)=\sum_{x_{i}}\left\{q\left(x_{i}\right)e^{-q(x_{i})}-p\left(x_{i}\right)e^{-p(x_{i})}\right\}. \end{array}$$

Note that Definition 2 characterizes a kind of relationship between two distributions from the perspective of information theory. In fact, this is a reasonable information measure that focuses on the effects of small probability elements regarded as message importance for two end-to-end distributions. On one hand, the MITM provides a tool to reflect the change of message importance in the whole transfer process. On the other hand, it also reveals the entire information feature variation of two end-to-end distributions, which we can use as a promising tool in the data mining.

### 1.2. Related Works for Information Measures in Big Data

There exist a variety of different information measures handling the problem of distributions, which can play a crucial role in many applications involved with artificial intelligence as well as big data analysis and processing.

As typical information measures, Shannon entropy and Renyi entropy are applicable to texture classification, intrinsic dimension estimation [19]. As well, the relative entropy, a kind of K-L divergence, is suitable for outlier detection [20] and functional magnetic resonance imaging (FMRI) data processing [21]. Moreover, the MIM and non-parametric message importance measure (NMIM) both focusing on the small probability events, have been proven effective in anomaly detection [17,22,23]. What is more, information divergences such as message importance (M-I) divergence can be applicable to extending methods of machine learning by using distributions and their relationship as features [24].

In addition, some information measures are proposed to reveal the correlation of message during the information transfer process. For example, the directed information [25,26,27,28] and Schreiber’s transfer entropy [29] are commonly applied to infer the causality structure and characterize the information transfer process. Moreover, referring to the idea from dynamical system theory, new information transfer measures are proposed to indicate the causality between states and control the systems [30,31,32].

However, in spite of numerous kinds of information measures, few works focus on how to characterize the information transfer from the perspective of message importance in big data. To this end, a new information measure different from the above is introduced.

### 1.3. Organization

We organize the rest of this paper as follows. In Section 2, we investigate the variation of message importance in the information transfer process by using MITM. In Section 3, we introduce the message importance transfer capacity measured by the MITM to describe the information transfer system with additive disturbance. In Section 4, the MITM and the KL divergence are used to guide the queue length selection for MEC from the viewpoint of the queue theory. Moreover, we also present some simulations to validate our theoretical results. Finally, we conclude in Section 5.

## 2. The Information Distance for Message Importance Variation

We now investigate the variation of message importance between two distributions by using an information transfer measure. This characterizes the information distance from the perspective of message importance, which can also reflect the robustness of the information transfer measure.

Consider an observation model, $P_{g_{0}|f_{0}}$: $f_{0}\left(x\right)\to g_{0}\left(x\right)$, namely an information transfer map for the variable *X* from one distribution $f_{0}\left(x\right)$ to the other distribution $g_{0}\left(x\right)$. In fact, it turns out to be not easy to cope with the two distributions. Instead, considering the similar way in [33], the relationship between $f_{0}\left(x\right)$ and $g_{0}\left(x\right)$ is given by

(8) $$g_{0}\left(x\right)=f_{0}\left(x\right)+\epsilon f_{0}^{\alpha }\left(x\right)u\left(x\right),$$

and the constraint condition satisfies

(9) $$\int_{S_{x}}\epsilon f_{0}^{\alpha }\left(x\right)u\left(x\right)dx=0,$$

where ϵ and α are two positive adjustable coefficients, as well as u(x) is a perturbation function of the variable *X* in the interval $S_{x}$.

Then, we discuss the information distance of message importance measured by the MITM in the Definition 2. This characterizes the difference between the origin and the destination of the information transfer from the viewpoint of message importance. By using the model $P_{g_{0}|f_{0}}$: $f_{0}\left(x\right)\to g_{0}\left(x\right)$ mentioned above, the end-to-end MITM is investigated in the information transfer process as follows.

**Proposition** **1.** *For two probability distributions $g_{0}\left(x\right)$ and $f_{0}\left(x\right)$ whose relationship satisfies the conditions Equations (8) and (9), the MITM is given by*

(10) $$\begin{array}{c} D_{I}(g_{0}\left(x\right)||f_{0}\left(x\right))\\ =\int_{S_{x}}\left\{g_{0}\left(x\right)e^{-g_{0}\left(x\right)}-f_{0}\left(x\right)e^{-f_{0}\left(x\right)}\right\}dx\\ =\epsilon \sum_{i=1}^{\infty }\frac{{\left(-1\right)}^{i}\left(i+1\right)}{i!}\int_{S_{x}}f_{0}^{i+\alpha }\left(x\right)u\left(x\right)dx\\ +\frac{\epsilon^{2}}{2}\sum_{i=1}^{\infty }\frac{{\left(-1\right)}^{i}\left(i+1\right)}{(i-1)!}\int_{S_{x}}f_{0}^{i-1+2\alpha }\left(x\right)u^{2}\left(x\right)dx+o\left(\epsilon^{2}\right), \end{array}$$

*where ϵ and α are parameters, u(x) denotes a function of the variable X, and $|D_{I}(g_{0}\left(x\right)||f_{0}\left(x\right))|\leq \int_{S_{x}}\left|\epsilon f_{0}^{\alpha }\left(x\right)u\left(x\right)\right|dx$ that satisfies the constraint Equation (5).*

**Proof** **of** **Proposition** **1.** According to the Binomial theorem, it is not difficult to see that

(11) $$\begin{array}{c} g_{0}^{i}\left(x\right)-f_{0}^{i}\left(x\right)\\ ={\left[f_{0}\left(x\right)+\epsilon f_{0}^{\alpha }\left(x\right)u\left(x\right)\right]}^{i}-f_{0}^{i}\left(x\right)\\ =\sum_{r=1}^{i}C_{i}^{r}f_{0}^{i-r}\left(x\right){\left[\epsilon f_{0}^{\alpha }\left(x\right)u\left(x\right)\right]}^{r}. \end{array}$$

Then, by using Taylor series expansion of $e^{x}$, it is readily seen that

(12) $$\begin{array}{c} e^{-g_{0}\left(x\right)}-e^{-f_{0}\left(x\right)}\\ =\sum_{i=0}^{\infty }\frac{{\left(-1\right)}^{i}}{i!}\left[g_{0}^{i}\left(x\right)-f_{0}^{i}\left(x\right)\right]\\ =\epsilon \sum_{i=1}^{\infty }\frac{{\left(-1\right)}^{i}}{(i-1)!}f_{0}^{\left(i-1+\alpha \right)}\left(x\right)u\left(x\right)+\frac{\epsilon^{2}}{2}\sum_{i=2}^{\infty }\frac{{\left(-1\right)}^{i}}{(i-2)!}f_{0}^{i-2+2\alpha }\left(x\right)u^{2}\left(x\right)+o\left(\epsilon^{2}\right). \end{array}$$

Therefore, by substituting Equation (12) into Equation (6), the proof of the proposition can be readily completed. ☐

Furthermore, it is not difficult to gain the MITM between the two different distributions $g_{1}^{\left(u\right)}$ and $g_{2}^{\left(u\right)}$ based on the same reference distribution $f_{0}\left(x\right)$ as follows

(13) $$\begin{array}{c} D_{I}(g_{1}^{\left(u\right)}\left(x\right)||g_{2}^{\left(u\right)}\left(x\right))\\ =\left[L\left(g_{1}^{\left(u\right)}\left(x\right)\right)-L\left(f_{0}\left(x\right)\right)\right]-\left[L\left(g_{2}^{\left(u\right)}\left(x\right)\right)-L\left(f_{0}\left(x\right)\right)\right]\\ =\epsilon \sum_{i=1}^{\infty }\frac{{\left(-1\right)}^{i}\left(i+1\right)}{i!}\int_{S_{x}}f_{0}^{i+\alpha }\left(x\right)\left[u_{1}\left(x\right)-u_{2}\left(x\right)\right]dx\\ +\frac{\epsilon^{2}}{2}\sum_{i=1}^{\infty }\frac{{\left(-1\right)}^{i}\left(i+1\right)}{(i-1)!}\int_{S_{x}}f_{0}^{i-1+2\alpha }\left(x\right)\left[u_{1}^{2}\left(x\right)-u_{2}^{2}\left(x\right)\right]dx+o\left(\epsilon^{2}\right), \end{array}$$

where

(14a) $$g_{1}^{\left(u\right)}\left(x\right)=f_{0}\left(x\right)+\epsilon f_{0}^{\alpha }\left(x\right)u_{1}\left(x\right),\;\forall x\in S_{x},$$

(14b) $$g_{2}^{\left(u\right)}\left(x\right)=f_{0}\left(x\right)+\epsilon f_{0}^{\alpha }\left(x\right)u_{2}\left(x\right),\;\forall x\in S_{x},$$

in which the ϵ and α are parameters, $u_{1}\left(x\right)$ and $u_{2}\left(x\right)$ denote functions of the variable *X* in the interval $S_{x}$, and $|D_{I}(g_{1}\left(x\right)||g_{2}\left(x\right))|\leq \int_{S_{x}}\left|\epsilon f_{0}^{\alpha }\left(x\right)\left\{u_{1}\left(x\right)-u_{2}\left(x\right)\right\}\right|dx$.

Similarly, it is available for the discrete probability distributions to have the same form of MITM as that mentioned in the Proposition 1. In particular, for two distributions $Q_{0}=\left\{q_{0}\left(x_{1}\right),q_{0}\left(x_{2}\right),\ldots ,q_{0}\left(x_{n}\right)\right\}$ and $P_{0}=\{p_{0}\left(x_{1}\right),$$p_{0}\left(x_{2}\right),\ldots ,p_{0}\left(x_{n}\right)\}$, it is easy to see that if the relationship between $Q_{0}$ and $P_{0}$ satisfies

(15) $$q_{0}\left(x_{i}\right)=p_{0}\left(x_{i}\right)+\epsilon p_{0}^{\alpha }\left(x_{i}\right)\tilde{u}\left(x_{i}\right),$$

with the constraint condition $\sum_{x_{i}}p_{0}^{\alpha }\left(x_{i}\right)\tilde{u}\left(x_{i}\right)=0$, we will have

(16) $$\begin{array}{c} D_{I}(Q_{0}\left|\right|P_{0})\\ =\epsilon \sum_{i=1}^{\infty }\frac{{\left(-1\right)}^{i}\left(i+1\right)}{i!}\sum_{x_{i}}p_{0}^{i+\alpha }\left(x_{i}\right)\tilde{u}\left(x_{i}\right)+\frac{\epsilon^{2}}{2}\sum_{i=1}^{\infty }\frac{{\left(-1\right)}^{i}\left(i+1\right)}{(i-1)!}\sum_{x_{i}}p_{0}^{i-1+2\alpha }\left(x_{0}\right){\tilde{u}}^{2}\left(x_{0}\right)+o\left(\epsilon^{2}\right), \end{array}$$

where ϵ and α are adjustable coefficients, and $\tilde{u}\left(x_{i}\right)$ is a perturbation function of the variable *X*. Moreover, it is not difficult to gain the discrete form of Equation (13) in the same way as above.

**Remark** **1.** *By resorting to the information distance measured by the MITM, the message importance distinction between two different distributions can be characterized. In the observation model mentioned in Equation (8), it is apparent that the parameter ϵ dominates the information distance when the perturbation function is finite and the parameter α<∞. Furthermore, the MITM is convergent with the order of O(ϵ) in the case of small parameter ϵ. Actually, it provides a way to apply MITM to measure the message importance variation in an information transfer process.*

## 3. Message Importance Transfer Capacity

**Figure 1:**
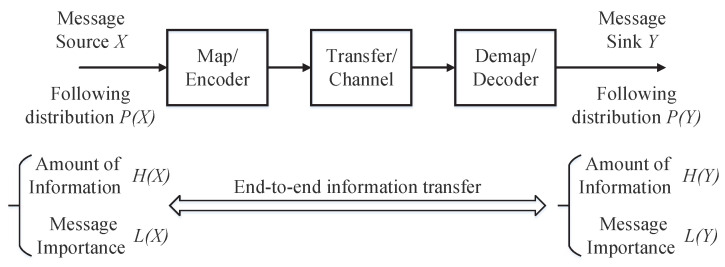
Information transfer system model.

In this section, we shall utilize the MITM to analyze the information transfer processing shown in Figure 1. To this end, we propose the message importance transfer capacity based on the MITM as follows.

**Definition** **3.** *Assume that there exists an information transfer process*

(17) $$\begin{array}{c} \{X,p(y|x),Y\}, \end{array}$$

*where the p(y|x) is a probability distribution matrix characterizing the information transfer from the variable X to Y. The message importance transfer capacity is defined as*

(18) $$\begin{array}{cc} & C=\max_{p(x)}\left\{L\left(Y\right)-L\left(Y|X\right)\right\}, \end{array}$$

*where $p\left(y\right)=\int_{S_{x}}p\left(x\right)p\left(y|x\right)dx$, $L\left(Y\right)=\int_{S_{y}}p\left(y\right)e^{-p(y)}dy$, and $L\left(Y|X\right)=\int_{S_{x}}\int_{S_{y}}p\left(xy\right)e^{-p(y|x)}dxdy$ with the constraint $\left|L\left(Y\right)-L\left(Y|X\right)\right|\leq {\lambda ∥p\left(y\right)-p\left(y|x\right)∥}_{1}$.*

Then, we discuss some specific information transfer scenarios to have an insight into the applications of message importance transfer capacity, as follows.

### 3.1. Binary Symmetric Information Transfer Matrix

Consider the binary symmetric information transfer matrix, in which the original variables are complemented with the transfer probability. In particular, the rows of the probability matrix are permutations of each other and so are columns which can be seen in the following proposition.

**Proposition** **2.** *Assume an information transfer process {X,p(y|x),Y}, whose the information transfer matrix is described as*

(19) $$\begin{array}{c} p\left(y|x\right)=\left[\begin{array}{cc} 1-\beta & \beta \\ \beta & 1-\beta \end{array}\right], \end{array}$$

*which implies that the variable X and Y both follow binary distributions. In this case, we have the message importance transfer capacity as follows*

(20) $$\begin{array}{c} C\left(\beta \right)=e^{-\frac{1}{2}}-L\left(\beta \right), \end{array}$$

*where $L\left(\beta \right)=\beta e^{-\beta }+\left(1-\beta \right)e^{-(1-\beta )}$ with 0<β<1, and $\left|C\left(\beta \right)\right|\leq {\lambda ∥p\left(y\right)-p\left(y|x\right)∥}_{1}$ with $\lambda \geq \frac{e^{-\frac{1}{2}}-\beta e^{-\beta }+\left(1-\beta \right)e^{-(1-\beta )}}{\left|1-2\beta \right|}$.*

**Proof** **of** **Proposition** **2.** Assume that the distribution of variable *X* is a binary distribution (p,1−p). As well, it is readily seen that

(21) $$\begin{array}{cc} L(Y|X) & =\sum_{i=1}^{2}p\left(x_{i}\right)\sum_{y}p\left(y|x_{i}\right)e^{-p(y|x_{i})}\\ & =\sum_{y}p\left(y|x_{i}\right)e^{-p(y|x_{i})}\\ & =\beta e^{-\beta }+\left(1-\beta \right)e^{-(1-\beta )}=L\left(\beta \right). \end{array}$$

Moreover, according to the definition of *C* in Equation (18), we have

(22) $$\begin{array}{c} C(p,\beta )\\ =\max_{p}\left\{\left[p+\beta \left(1-2p\right)\right]e^{-[p+\beta (1-2p)]}+\left[\left(1-p\right)+\beta \left(2p-1\right)\right]e^{-[(1-p)+\beta (2p-1)]}\right\}-L\left(\beta \right). \end{array}$$

Then, it is not difficult to see that

(23) $$\begin{array}{c} \frac{\partial C(p,\beta )}{\partial p}\\ =\left(1-2\beta \right)\left\{\left[1-p-\beta \left(1-2p\right)\right]e^{-[p+\beta (1-2p)]}-\left[1-\left(1-p\right)-\beta \left(2p-1\right)\right]e^{-[(1-p)+\beta (2p-1)]}\right\}. \end{array}$$

According to the monotonically decreasing of $\frac{\partial C(p,\beta )}{\partial p}$ for p∈[0,1], it is readily seen that $p=\frac{1}{2}$ is the only solution for $\frac{\partial C(p,\beta )}{\partial p}=0$. Therefore, by substituting $p=\frac{1}{2}$ into C(p,β), the proposition is testified. ☐

**Remark** **2.** *In light of Proposition 2, on one hand, when β=1/2, in other words, there is just random information transfer process, we will obtain the lower bound of the message importance transfer capacity that is C(β)=0. On the other hand, when β=0, namely, the information transfer process is definite, we will gain the maximum message importance transfer capacity.*

### 3.2. Binary Erasure Information Transfer Matrix

The binary erasure information transfer matrix is similar to the binary symmetric one, however, in the former a part of information is lost rather than corrupted. In other words, a fraction of information is erased. In this case, the message importance transfer capacity is discussed as follows.

**Proposition** **3.** *Consider an information transfer process {X,p(y|x),Y}, in which the information transfer matrix is described as*

(24) $$\begin{array}{c} p\left(y|x\right)=\left[\begin{array}{ccc} 1-\beta & 0 & \beta \\ 0 & 1-\beta & \beta \end{array}\right], \end{array}$$

*which indicates that X follows the binary distribution and Y follows the 3-ary distribution. Then, we have*

(25) $$\begin{array}{c} C\left(\beta \right)=\left(1-\beta \right)e^{-\frac{1}{2}\left(1-\beta \right)}+\beta e^{-\beta }-L\left(\beta \right), \end{array}$$

*where $L\left(\beta \right)=\beta e^{-\beta }+\left(1-\beta \right)e^{-(1-\beta )}$ with 0<β<1 and $\left|C\left(\beta \right)\right|\leq {\lambda ∥p\left(y\right)-p\left(y|x\right)∥}_{1}$ with $\lambda \geq e^{-\frac{1}{2}\left(1-\beta \right)}-e^{-(1-\beta )}$.*

**Proof** **of** **Proposition** **3.** Assume the distribution of variable *X* is (p,1−p). As well, according to the binary erasure information transfer matrix, it is not difficult to see that

(26) $$\begin{array}{cc} C(p,\beta ) & =\max_{p}\left\{p\left(1-\beta \right)e^{-p(1-\beta )}+\beta e^{-\beta }+\left(1-p\right)\left(1-\beta \right)e^{-(1-p)(1-\beta )}\right\}-L\left(\beta \right), \end{array}$$

where $L\left(\beta \right)=\beta e^{-\beta }+\left(1-\beta \right)e^{-(1-\beta )}$. Then, we have

(27) $$\begin{array}{cc} \frac{\partial C(p,\beta )}{\partial p} & =\left(1-\beta \right)\left\{\left[1-\left(1-\beta \right)p\right]e^{-(1-\beta )p}-\left[1-\left(1-\beta \right)\left(1-p\right)\right]e^{-(1-\beta )(1-p)}\right\}. \end{array}$$

Due to the monotonically decreasing of $\frac{\partial C(p,\beta )}{\partial p}$ for p∈[0,1], it is readily seen that p=1/2 is the only solution for $\frac{\partial C(p,\beta )}{\partial p}=0$. Thus, by substituting p=1/2 into Equation (26), the proposition is readily verified. ☐

### 3.3. Strongly Symmetric Information Transfer Matrix

In terms of the strongly symmetric information transfer matrix, it can be regarded as an extension of the binary symmetric one. The message information transfer capacity of the former is also analogous to the that of the latter, which is discussed as follows.

**Proposition** **4.** *Assume an information transfer process with the strongly symmetric transfer matrix as follows*

(28) $$\begin{array}{c} p\left(y|x\right)=\left[\begin{array}{cccc} 1-\beta & \frac{\beta }{K-1} & \ldots & \frac{\beta }{K-1}\\ \frac{\beta }{K-1} & 1-\beta & \ldots & \frac{\beta }{K-1}\\ \ldots & \ldots & \ldots & \ldots \\ \frac{\beta }{K-1} & \ldots & \frac{\beta }{K-1} & 1-\beta \end{array}\right], \end{array}$$

*which implies that the variable X and Y both obey K-ary distribution. We have*

(29) $$\begin{array}{c} C\left(\beta \right)=e^{-\frac{1}{K}}-\left\{\left(1-\beta \right)e^{-(1-\beta )}+\beta e^{-\frac{\beta }{K-1}}\right\}, \end{array}$$

*where the parameter β∈(0,1) and $\left|C\left(\beta \right)\right|\leq {\lambda ∥p\left(y\right)-p\left(y|x\right)∥}_{1}$ with $\lambda \geq \frac{e^{-1/K}-\left(1-\beta \right)e^{-(1-\beta )}-\beta e^{-\beta /K-1}}{2|1-\beta -1/K|}$.*

**Proof** **of** **Proposition** **4.** Assume the probability distribution of variable *X* is $\{p\left(x_{1}\right),p\left(x_{2}\right),\ldots ,$$p(x_{K})\}$. As for the strongly symmetric transfer matrix, when the probabilities of $x_{i}$ are equal, that is, $p\left(x_{1}\right)=p\left(x_{2}\right)=\ldots =p\left(x_{K}\right)=1/K$, we will have

(30) $$\begin{array}{cc} p(y_{j}) & =\sum_{i=1}^{K}p\left(x_{i},y_{j}\right)=\sum_{i=1}^{K}p\left(x_{i}\right)p\left(y_{j}|x_{i}\right)\\ & =\frac{1}{K}\sum_{i=1}^{K}p\left(y_{j}|x_{i}\right)=\frac{1}{K}, \end{array}$$

which indicates that the probabilities of $y_{j}$ (j=1,2,…,K) are equal.

In addition, on account of the information transfer matrix, it is easy to see that

(31) $$\begin{array}{cc} L(Y|X) & =\sum_{i=1}^{2}p\left(x_{i}\right)\sum_{y_{j}}p\left(y_{j}|x_{i}\right)e^{-p(y_{j}|x_{i})}\\ & =\sum_{y_{j}}p\left(y_{j}|x_{i}\right)e^{-p(y_{j}|x_{i})}\\ & =\beta e^{-\frac{\beta }{K-1}}+\left(1-\beta \right)e^{-(1-\beta )}. \end{array}$$

What is more, according to the definition of message importance transfer capacity in Equation (18), it is readily seen that

(32) $$\begin{array}{cc} C(\beta ) & =\max_{p(x)}\left\{L\left(Y\right)\right\}-\left[\beta e^{-\frac{\beta }{K-1}}+\left(1-\beta \right)e^{-(1-\beta )}\right], \end{array}$$

where $L\left(Y\right)=\sum_{y_{j}}p\left(y_{j}\right)e^{-p(y_{j})}$.

Then, by using Lagrange multiplier method, we have

(33) $$\begin{array}{cc} G(p\left(y_{j}\right),\lambda_{0}) & =\sum_{y_{j}}p\left(y_{j}\right)e^{-p(y_{j})}+\lambda_{0}[\sum_{y_{j}}p\left(y_{j}\right)-1]. \end{array}$$

By setting $\frac{\partial G(p\left(y_{j}\right),\lambda_{0})}{\partial p(y_{j})}=0$ and $\frac{\partial G(p\left(y_{j}\right),\lambda_{0})}{\partial \lambda_{0}}=0$, it can be readily verified that the extreme value of $\sum_{y_{j}}p\left(y_{j}\right)e^{-p(y_{j})}$ is achieved by the solution $p\left(y_{1}\right)=p\left(y_{2}\right)=\ldots =p\left(y_{K}\right)=1/K$.

In light of $\frac{\partial^{2}G\left(p\left(y_{j}\right),\lambda_{0}\right)}{\partial p^{2}\left(y_{j}\right)}<0$ with respect to $p\left(y_{j}\right)\in \left[0,1\right]$, it is readily seen that when the variable *X* follows the uniform distribution which leads to the uniform distribution for variable *Y*, we will gain the message importance transfer capacity C(β). Then, it is easy for us to complete the proof of the proposition. ☐

### 3.4. Continuous Case for the Message Importance Transfer Capacity

By using the MITM as a measuring tool, the information transfer process in the continuous case is investigated. Considering the information transfer process described as Equation (17), it is significant to clarify the effect of the continuous disturbance on the message importance transfer capacity.

**Theorem** **1.** *Assume that there exists an information transfer process between the variable X and Y, denoted by {X,p(y|x),Y}, where E[X]=0, $E\left[X^{2}\right]=P_{s}$, Y=X+Z. The variable Z denotes an independent memoryless additive disturbance, whose mean and variance satisfy that E[Z]=μ and $E\left[{\left(Z-\mu \right)}^{2}\right]=\sigma^{2}$, respectively. Then, we adopt the MITM to measure the message importance transfer capacity as*

(34a) $$\begin{array}{c} \begin{array}{cc} C(P_{s}) & =\max_{p(x)}D_{I}\left(Y||Z\right)\\ & =\max_{p(x)}\left\{L\left(Y\right)\right\}-L\left(Z\right)\\ & =\max_{p(x)}\left\{\int_{-\infty }^{+\infty }p\left(y\right)e^{-p(y)}dy\right\}-\sum_{j=0}^{\infty }\frac{{\left(-1\right)}^{j}}{j!}\int_{-\infty }^{\infty }p^{j+1}\left(z\right)dz, \end{array} \end{array}$$

(34b) $$\begin{array}{c} \begin{array}{cc} & s.t.\;E\left[Y^{2}\right]=P_{s}+P_{N}, \end{array} \end{array}$$

*where $P_{N}=\mu^{2}+\sigma^{2}$, $p\left(y\right)=\int_{S_{x}}p\left(x\right)p\left(y|x\right)dx$ with the constraint $\left|L\left(Y\right)-L\left(Z\right)\right|\leq {\lambda ∥p\left(y\right)-p\left(z\right)∥}_{1}$ (λ>0 is the Lipschitz constant), and L(·) is the MIM operator. That is, the variance of X makes more effect on the constraint of the message importance transfer capacity.*

**Proof** **of** **Theorem** **1.** According to Equation (17), we have

(35) $$\left\{\begin{array}{c} x(x,y)=x\\ z(x,y)=y-x. \end{array}\right.$$

Then, it is not difficult to see that

(36) $$\begin{array}{c} p\left(xy\right)=p\left(xz\right)|J\left(\frac{xz}{xy}\right)|=p\left(xz\right)\left|\begin{array}{cc} \frac{\partial x}{\partial x} & \frac{\partial x}{\partial y}\\ \frac{\partial z}{\partial x} & \frac{\partial z}{\partial y} \end{array}\right|=p\left(xz\right). \end{array}$$

Moreover, by virtue of the independence of *X* and *Z*, we have

(37) $$\begin{array}{c} p(x)p(y|x)=p(x)p(z), \end{array}$$

which indicates that

(38) $$\begin{array}{c} p(y|x)=p(z). \end{array}$$

Then, we have

(39) $$\begin{array}{cc} L(Y|X) & =\int_{x}\int_{y}p\left(x\right)p\left(y|x\right)e^{-p(y|x)}dxdy\\ & =\int_{x}\int_{z}p\left(x\right)p\left(z\right)e^{-p(z)}dxdz\\ & =\int_{x}p\left(x\right)\left\{\int_{z}p\left(z\right)e^{-p(z)}dz\right\}dx=L\left(Z\right). \end{array}$$

Consequently, in terms of the Definition 3, it is readily seen that L(Y)−L(Y|X) can be written as L(Y)−L(Z), which testifies Equation (34a).

Furthermore, according to the fact that $E\left[Y^{2}\right]=E\left[{\left(X+Z\right)}^{2}\right]=E\left[X^{2}\right]+E\left[Z^{2}\right]=P_{s}+P_{N}$, we have the constraint condition Equation (34b). As well, by substituting the definition of MITM into Equation (34a), the Theorem 1 is proved. ☐

**Remark** **3.** *For the message importance transfer capacity with an additive disturbance, it is worth noting that the distribution of the transferred variable Y with the constrained variance may have a significant impact on the practical applications. In practice, the variance can be regarded as the power of signals. Consequently, the message importance transfer capacity mentioned in Theorem 1 can be used to guide the signal transfer process with additive disturbance, if the system does not have relatively large change.*

**Corollary** **1.** *Consider an information transfer process {X,p(y|x),Y}, where Y=X+Z and the variable Z denotes an independent Gaussian disturbance with $E\left[Z\right]=\mu_{z}$ and $E\left[Z^{2}\right]=\sigma_{z}^{2}$. Assume that the variable X follows a Gaussian mixture model as*

(40) $$\begin{array}{c} P_{X}\left(x\right)=\frac{1}{N}\sum_{k=1}^{k=N}\phi \left(x|\mu_{k},\sigma_{k}^{2}\right), \end{array}$$

*where $\mu_{k}$ and $\sigma_{k}^{2}$ are the means and the variances of independent Gaussian distributions, in other words, $\phi \left(x|\mu_{k},\sigma_{k}^{2}\right)=1/\left(\sqrt{2\pi }\sigma_{k}\right)\exp\left\{-{\left(x-\mu_{k}\right)}^{2}/\left(2\sigma_{k}^{2}\right)\right\}$. In this case, the message importance transfer capacity $C(\mu_{x},\sigma_{x}^{2})$ with the constraint $|C\left(\mu_{x},\sigma_{x}^{2}\right)|\leq \lambda ∥P_{Y}\left(y\right)-P_{Z}\left(z\right){∥}_{1}$, is*

(41) $$\begin{array}{cc} C(\mu_{x},\sigma_{x}^{2}) & =\max_{P_{X}\left(x\right)}D_{I}\left(Y||Z\right)\\ & ≐\frac{1}{2\sqrt{\pi \sigma_{z}^{2}}}-\frac{1}{2N^{2}\sqrt{\pi (\Theta +\sigma_{z}^{2})}}\sum_{i=1}^{N}\sum_{j=1}^{N}e^{-\frac{{\left(\mu_{i}-\mu_{j}\right)}^{2}}{4(\Theta +\sigma_{z}^{2})}}, \end{array}$$

*where $\Theta =\frac{1}{N}\sum_{k=1}^{N}\sigma_{k}^{2}$. In particular, the parameters $\sigma_{k}^{2}$ can be controlled by the parameters $\sigma_{x}^{2}$, $\mu_{x}$ and $\mu_{k}$ in a system, where the $\mu_{x}$ and $\sigma_{x}^{2}$ are the mean and variance of the variable X, which are given by*

(42a) $$\begin{array}{c} \begin{array}{c} \mu_{x}=\frac{1}{N}\sum_{k=1}^{N}\mu_{k}, \end{array} \end{array}$$

(42b) $$\begin{array}{c} \begin{array}{cc} \sigma_{x}^{2} & =\frac{1}{N}\sum_{k=1}^{N}\left(\sigma_{k}^{2}+\mu_{k}^{2}\right)-{\left(\frac{1}{N}\sum_{k=1}^{N}\mu_{k}\right)}^{2}. \end{array} \end{array}$$

**Proof** **of** **Corollary** **1.** As for the Gaussian variable *Z* satisfying $E\left[Z\right]=\mu_{z}$ and $E\left[Z^{2}\right]=\sigma_{z}^{2}$, the DMIM is given by

(43) $$\begin{array}{cc} L(Z) & =\int_{-\infty }^{\infty }\frac{1}{\sqrt{2\pi \sigma_{z}^{2}}}e^{-\frac{{\left(z-\mu_{z}\right)}^{2}}{2\sigma_{z}^{2}}}e^{-\frac{1}{\sqrt{2\pi \sigma_{0}^{2}}}e^{-\frac{{\left(z-\mu_{z}\right)}^{2}}{2\sigma_{z}^{2}}}}dz\\ & =\sum_{i=0}^{\infty }\frac{{\left(-1\right)}^{i}}{i!{\left(\sqrt{2\pi \sigma_{z}^{2}}\right)}^{i+1}}\frac{1}{2}\sqrt{\frac{\pi }{\alpha_{0}}}e^{\frac{\beta_{0}^{2}-\alpha_{0}\gamma_{0}}{\alpha_{0}}}\cdot erf\left(\sqrt{\alpha_{0}}z+\frac{\beta_{0}}{\sqrt{\alpha_{0}}}\right){|}_{z=-\infty }^{z=\infty }, \end{array}$$

where the erf(·) is the error function, namely,

(44) $$\begin{array}{c} erf\left(z\right)=\frac{2}{\sqrt{\pi }}\int_{0}^{z}e^{-t^{2}}dt, \end{array}$$

and the parameters $\alpha_{0}$, $\beta_{0}$ and $\gamma_{0}$ satisfy

(45a) $$\begin{array}{c} \begin{array}{c} \alpha_{0}=\frac{i+1}{2\sigma_{z}^{2}}, \end{array} \end{array}$$

(45b) $$\begin{array}{c} \begin{array}{c} \beta_{0}=-\frac{\left(i+1\right)\mu_{z}}{2\sigma_{z}^{2}}, \end{array} \end{array}$$

(45c) $$\begin{array}{c} \begin{array}{c} \gamma_{0}=\frac{\left(i+1\right)\mu_{z}^{2}}{2\sigma_{z}^{2}}. \end{array} \end{array}$$

Then, it is readily seen that

(46) $$\begin{array}{cc} L(Z) & =\sum_{i=0}^{\infty }\frac{{\left(-1\right)}^{i}}{i!\sqrt{i+1}{\left(\sqrt{2\pi \sigma_{z}^{2}}\right)}^{i}}, \end{array}$$

which can be approximated by

(47) $$L\left(Z\right)≐1-\frac{1}{2\sqrt{\pi \sigma_{z}^{2}}}.$$

In addition, according to Y=X+Z (with the independent *X* and *Z*), it is readily seen that the variable *Y* also follows a Gaussian mixture model as

(48) $$\begin{array}{c} P_{Y}\left(y\right)\\ =\int_{-\infty }^{\infty }P_{X}\left(x\right)P_{Z}\left(y-x\right)dx\\ =\frac{1}{N}\sum_{k=1}^{k=N}\int_{-\infty }^{\infty }\phi \left(x|\mu_{k},\sigma_{k}\right)\frac{1}{\sqrt{2\pi \sigma_{z}^{2}}}e^{-\frac{{\left(y-x-\mu_{z}\right)}^{2}}{2\sigma_{z}^{2}}}dx\\ =\frac{1}{N}\sum_{k=1}^{k=N}\frac{1}{\sqrt{2\pi (\sigma_{k}^{2}+\sigma_{z}^{2})}}e^{-\frac{{\left(y-\mu_{k}-\mu_{z}\right)}^{2}}{2(\sigma_{z}^{2}+\sigma_{k}^{2})}}\\ =\frac{1}{N}\sum_{k=1}^{k=N}\phi \left(y|{\tilde{\mu }}_{k},{\tilde{\sigma }}_{k}^{2}\right), \end{array}$$

where ${\tilde{\mu }}_{k}=\mu_{k}+\mu_{z}$ and ${\tilde{\sigma }}_{k}^{2}=\sigma_{k}^{2}+\sigma_{z}^{2}$ (k=1,2,…,N).

By using of Taylor series extension, we have the DMIM of variable *Y* as follows

(49) $$\begin{array}{cc} L(Y) & =\int_{-\infty }^{\infty }P_{Y}\left(y\right)e^{-P_{Y}\left(y\right)}dy\\ & =\int_{-\infty }^{\infty }P_{Y}\left(y\right)\left[1-P_{Y}\left(y\right)+O\left(P_{Y}^{2}\left(y\right)\right)\right]dy\\ & ≐1-\int_{-\infty }^{\infty }P_{Y}^{2}\left(y\right)dy. \end{array}$$

Then, according to Equation (48), it is readily seen that

(50) $$\begin{array}{cc} L(Y) & ≐1-\frac{1}{N^{2}}\sum_{i=1}^{N}\sum_{j=1}^{N}\int_{-\infty }^{\infty }\phi \left(y|{\tilde{\mu }}_{i},{\tilde{\sigma }}_{i}^{2}\right)\phi \left(y|{\tilde{\mu }}_{j},{\tilde{\sigma }}_{j}^{2}\right)dy\\ & =1-\frac{1}{N^{2}}\sum_{i=1}^{N}\sum_{j=1}^{N}\frac{1}{2\pi \sqrt{\sigma_{i}^{2}\sigma_{j}^{2}}}\left\{\frac{1}{2}\sqrt{\frac{\pi }{\alpha_{1}}}e^{\frac{\beta_{1}^{2}-\alpha_{1}\gamma_{1}}{\alpha_{1}}}\cdot erf\left(\sqrt{\alpha_{1}}y+\frac{\beta_{1}}{\sqrt{\alpha_{1}}}\right){|}_{y=-\infty }^{y=\infty }\right\}, \end{array}$$

where ${\tilde{\mu }}_{k}=\mu_{k}+\mu_{z}$ and ${\tilde{\sigma }}_{k}^{2}=\sigma_{k}^{2}+\sigma_{z}^{2}$ (k=1,2,…,N), the parameters $\alpha_{1}$, $\beta_{1}$ and $\gamma_{1}$ are

(51a) $$\begin{array}{c} \begin{array}{c} \alpha_{1}=\frac{{\tilde{\sigma }}_{i}^{2}+{\tilde{\sigma }}_{j}^{2}}{2{\tilde{\sigma }}_{i}^{2}{\tilde{\sigma }}_{j}^{2}}, \end{array} \end{array}$$

(51b) $$\begin{array}{c} \begin{array}{c} \beta_{1}=-\frac{{\tilde{\mu }}_{i}{\tilde{\sigma }}_{j}^{2}+{\tilde{\mu }}_{j}{\tilde{\sigma }}_{i}^{2}}{2{\tilde{\sigma }}_{i}^{2}{\tilde{\sigma }}_{j}^{2}}, \end{array} \end{array}$$

(51c) $$\begin{array}{c} \begin{array}{c} \gamma_{1}=\frac{{\tilde{\mu }}_{i}^{2}{\tilde{\sigma }}_{j}^{2}+{\tilde{\mu }}_{j}^{2}{\tilde{\sigma }}_{i}^{2}}{2{\tilde{\sigma }}_{i}^{2}{\tilde{\sigma }}_{j}^{2}}. \end{array} \end{array}$$

Then, it is not difficult to see that

(52) $$\begin{array}{c} L\left(Y\right)≐1-\frac{1}{N^{2}}\sum_{i=1}^{N}\sum_{j=1}^{N}\frac{1}{\sqrt{2\pi ({\tilde{\sigma }}_{i}^{2}+{\tilde{\sigma }}_{j}^{2})}}e^{-\frac{{\left({\tilde{\mu }}_{i}-{\tilde{\mu }}_{j}\right)}^{2}}{2({\tilde{\sigma }}_{i}^{2}+{\tilde{\sigma }}_{j}^{2})}}. \end{array}$$

where ${\tilde{\mu }}_{i}$ and ${\tilde{\sigma }}_{i}^{2}$ (or ${\tilde{\mu }}_{j}$ and ${\tilde{\sigma }}_{j}^{2}$) denote the means and the variances in Gaussian mixture model mentioned in Equation (48).

Furthermore, in the light of Equations (47) and (52), we have the message importance transfer measure with the constrained variances $\sigma_{k}^{2}$ as follows

(53a) $$\begin{array}{c} \begin{array}{cc} C(\mu_{x},\sigma_{x}^{2}) & =\max_{P(X)}\left\{L\left(Y\right)\right\}-L\left(Z\right)\\ & ≐\frac{1}{2\sqrt{\pi \sigma_{z}^{2}}}-\min_{P_{X}\left(x\right)}\left\{\frac{1}{N^{2}}\sum_{i=1}^{N}\sum_{j=1}^{N}\frac{e^{-\frac{{\left({\tilde{\mu }}_{i}-{\tilde{\mu }}_{j}\right)}^{2}}{2({\tilde{\sigma }}_{i}^{2}+{\tilde{\sigma }}_{j}^{2})}}}{\sqrt{2\pi ({\tilde{\sigma }}_{i}^{2}+{\tilde{\sigma }}_{j}^{2})}}\right\}. \end{array} \end{array}$$

(53b) $$\begin{array}{c} \begin{array}{cc} & s.t.\;\sum_{k=1}^{N}\sigma_{k}^{2}=N\Theta , \end{array} \end{array}$$

where the parameter Θ can be regarded as a constant which is controlled by the system parameters $\sigma_{x}^{2}$, $\mu_{x}$ and $\mu_{k}$, as follows

(54) $$\begin{array}{cc} \Theta & =\frac{1}{N}\sum_{k=1}^{N}\sigma_{k}^{2}=\sigma_{x}^{2}+\mu_{x}^{2}-\frac{1}{N}\sum_{k=1}^{N}\mu_{k}^{2}. \end{array}$$

Moreover, the parameter $\sigma_{z}^{2}$ is a system constant and $\mu_{k}$ are regarded as constants, while the parameters $\sigma_{k}^{2}$(k=1,2,…,N) can be adjusted flexibly. According to the Lagrange multiplier method, when $\sigma_{1}^{2}=\sigma_{2}^{2}=\ldots =\sigma_{N}^{2}=\Theta$, we have

(55) $$\begin{array}{c} \min_{P_{X}\left(x\right)}\left\{\frac{1}{N^{2}}\sum_{i=1}^{N}\sum_{j=1}^{N}\frac{e^{-\frac{{\left({\tilde{\mu }}_{i}-{\tilde{\mu }}_{j}\right)}^{2}}{2({\tilde{\sigma }}_{i}^{2}+{\tilde{\sigma }}_{j}^{2})}}}{\sqrt{2\pi ({\tilde{\sigma }}_{i}^{2}+{\tilde{\sigma }}_{j}^{2})}}\right\}=\frac{1}{2N^{2}\sqrt{\pi (\Theta +\sigma_{z}^{2})}}\sum_{i=1}^{N}\sum_{j=1}^{N}e^{-\frac{{\left(\mu_{i}-\mu_{j}\right)}^{2}}{4(\Theta +\sigma_{z}^{2})}}. \end{array}$$

By substituting Equation (55) into Equation (53a), the proof of Corollary 1 is already completed. ☐

**Figure 2:**
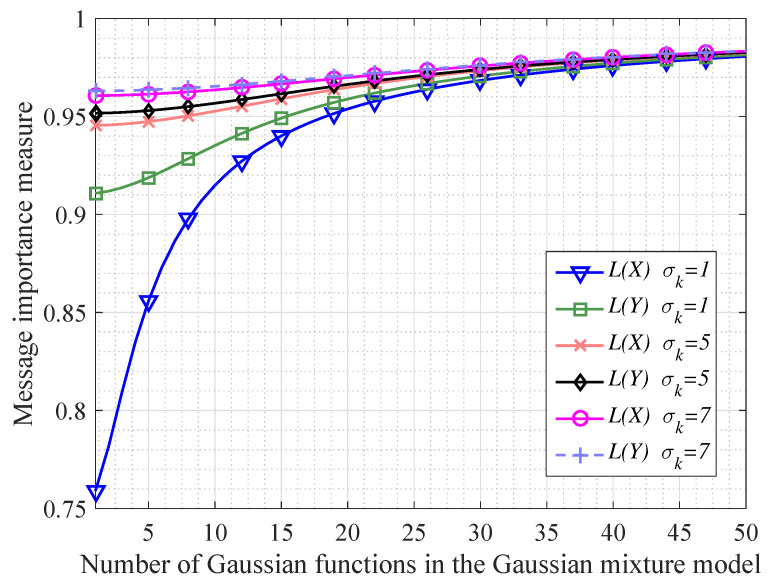
The comparison between the message importance measures for the original variable *X* and the final variable *Y* in an information transfer processing (where the variable *X* follows a Gaussian mixture model with all the variances of Gaussian functions as same as $\sigma_{k}^{2}$).

**Figure 3:**
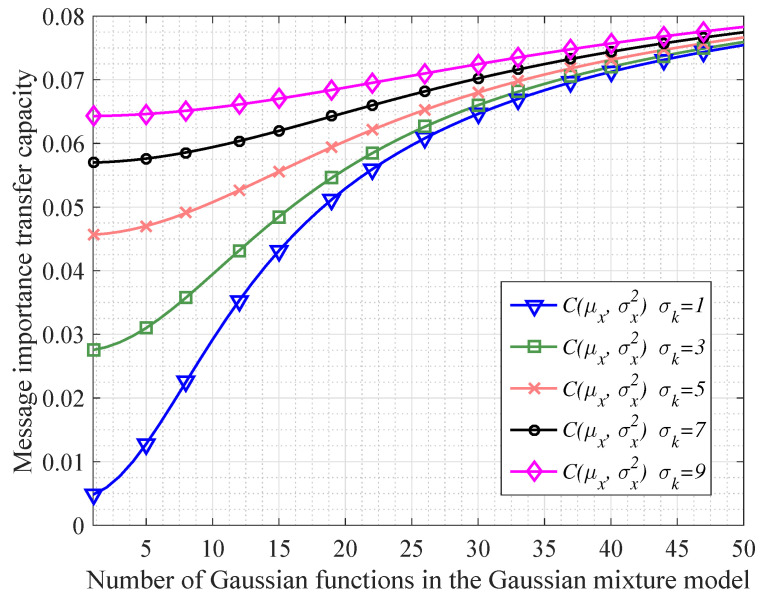
The performance of message importance transfer capacity in the Gaussian mixture model in which all the variances of Gaussian functions are the same as $\sigma_{k}^{2}$ ($\sigma_{k}=1,3,5,7,9$).

In order to investigate the continuous information transfer processing mentioned in Corollary 1, we do some simulations shown as Figure 2 and Figure 3. In particular, Figure 2 shows that when the variable *X* following a Gaussian mixture model transfers to the variable *Y*, the message importance measures of *X* and *Y* become more absolutely close with *N* increasing (*N* denotes the number of Gaussian functions in the Gaussian mixture model). Besides, we also see that the differences of message importance measures between the variable *X* and *Y* are not significant in the case of large variances $\sigma_{k}^{2}$. In addition, from Figure 3, it is seen that the message importance transfer capacity is increasing with the increment of the number of Gaussian functions. Moreover, the larger variances $\sigma_{k}^{2}$ of the Gaussian mixture model are, the larger message importance transfer capacity we have.

**Remark** **4.** *As for an additive disturbance system where the data source derive from a Gaussian mixture model, we can obtain the message importance transfer capacity, if there are all the same variances $\sigma_{k}^{2}$ for the Gaussian distribution components in the data source. In practice, when the power of signal source is controlled in a signal transfer processing, we can adjust signal distributions to achieve the optimal message importance transfer by using Corollary 1.*

## 4. Application in Mobile Edge Computing with the M/M/s/k Queue

As for mobile users, almost all of them have few computing resources and depend solely on cloud computing. This implies that the large distance between the cloud and the end devices is not suitable for the low delay requirement of the future applications. To cope with the issue, the MEC is proposed to improve cloud computing.

As far as the MEC is concerned, the edge servers are placed in the Base Stations (BSs) to reduce the delay, while context aware applications are close to the mobile users [34]. To characterize the MEC more specifically, a MEC model is constructed based on the queuing theory as follows.

**Figure 4:**
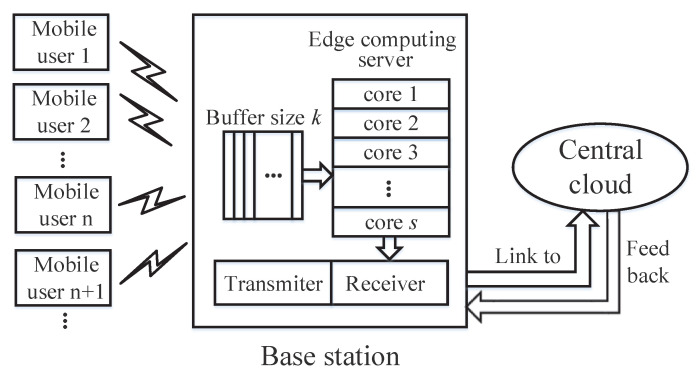
The queue model on the mobile edge computing system.

In terms of a MEC system in Figure 4, it consists of many mobile users, an edge server, and a central cloud located far from the local devices. For each mobile user, a part of or all the service requests can be offloaded to the corresponding edge server when the communication is disturbed by other mobile users or environmental noise. If the upper bound of the service rate for the edge server is larger than the sum of mobile users’ request rate, the offloaded requests will be coped with by the edge server. Otherwise, the overloaded requests will be offloaded to the central cloud for processing [35]. In these cases, the queue model on the edge server can be considered as the M/M/s/k queue, where the first *M* describes the request interarrival time of mobile users, the second *M* denotes the request service time in the edge server, and both of them follow exponential distribution; the parallel processing core number is *s*, which means each processing core can at most server one request simultaneously; the queuing buffer is *k* in the edge server. Note that we only consider a simple model on MEC to show the potential application of MITM. In fact, there may be some complicated cases in the MEC such as fault tolerance, failover, and the existence of overlay networks, etc.; we shall consider this in the near future.

In fact, it is significant for the MEC system to use the finite buffer size (or caching size) to approximate the infinite one, which can be treated as a problem of queue length selection. To do this, we exploit the MITM and KL divergence to measure the effect of queue states variation on the MEC performance as follows.

### 4.1. MITM in the Queueing Model

As a measurement for the distance of the message importance, MITM characterizes the difference between two distributions. This can be applied to distinguish the state probability distributions in queue models. To give more general analysis, we discuss the relationship between the queue state stationary distributions in the M/M/s/k model. The queue state stationary probability of the model with arrival rate $\tilde{\lambda }$ and service rate $\tilde{\mu }$ can be described as

(56a) $$\begin{array}{cc} & p_{0}={\left[\sum_{j=0}^{s-1}\frac{a^{j}}{j!}+\frac{a^{s}}{s!}\cdot \frac{1-\rho^{k+1}}{1-\rho }\right]}^{-1} \end{array}$$

(56b) $$\begin{array}{cc} & p_{j}=\frac{a^{j}}{j!}p_{0},\;\left(0<j<s\right) \end{array}$$

(56c) $$\begin{array}{cc} & p_{j}=\frac{a^{s}}{s!}\rho^{j-s}p_{0},\;\left(s\leq j\leq s+k\right), \end{array}$$

where *s* is the number of servers, *k* is the size of buffer or cache, the traffic intensity ρ=a/s<1 as well as $a=\tilde{\lambda }/\tilde{\mu }$.

Therefore, according to the definition 1, we can obtain the RMIM of the queue state stationary probability in the M/M/s/k model. Then, by use of Taylor series expansion, the approximate RMIM is given by

(57) $$\begin{array}{c} \sum_{j=0}^{s+k}p_{j}e^{-p_{j}}\\ =\sum_{j=0}^{s+k}p_{j}\left[1-p_{j}+O\left(p_{j}^{2}\right)\right]\\ ≐1-\sum_{j=0}^{s-1}\frac{{\left(\frac{a^{j}}{j!}\right)}^{2}}{{\left(\varphi_{1}+\varphi_{2}\frac{1-\rho^{k+1}}{1-\rho }\right)}^{2}}-\sum_{j=s}^{s+k}\frac{\varphi_{2}^{2}\rho^{2j}}{{\left(\varphi_{1}+\varphi_{2}\frac{1-\rho^{k+1}}{1-\rho }\right)}^{2}}\\ =1-p_{0}^{2}\left\{\sum_{j=0}^{s-1}{\left(\frac{a^{j}}{j!}\right)}^{2}+\frac{\varphi_{2}^{2}\left[1-\rho^{2(k+1)}\right]}{1-\rho^{2}}\right\}, \end{array}$$

where the parameter $\varphi_{1}$ and $\varphi_{2}$ are

(58a) $$\begin{array}{cc} & \varphi_{1}=\sum_{j=0}^{s-1}\frac{a^{j}}{j!}, \end{array}$$

(58b) $$\begin{array}{cc} & \varphi_{2}=\frac{a^{s}}{s!}. \end{array}$$

Furthermore, referring to Equation (57), we can use the MITM to characterize the information difference for the queue model as follows.

**Proposition** **5.** *In the M/M/s model, the MITM can be used to measure the information difference between two queue state probability stationary distributions $P_{k}=\left\{p_{k,0},p_{k,1},\ldots ,p_{k,s+k},0,0,\ldots ,0\right\}$ and $P_{k+1}=\{p_{k+1,0},$$p_{k+1,1},\ldots ,p_{k+1,s+k+1},0,\ldots ,0\}$ which are with buffer size k and k+1 respectively, as follows*

(59) $$\begin{array}{cc} D_{I}(P_{k+1}\left|\right|P_{k}) & =\sum_{j=0}^{s+k+1}p_{k+1,j}e^{-p_{k+1,j}}-\sum_{j=0}^{s+k}p_{j}e^{-p_{j}}\\ & ≐\left(\frac{1}{{\left(\varphi_{1}+\varphi_{2}\frac{1-\rho^{k+1}}{1-\rho }\right)}^{2}}-\frac{1}{{\left(\varphi_{1}+\varphi_{2}\frac{1-\rho^{k+2}}{1-\rho }\right)}^{2}}\right)\sum_{j=0}^{s-1}{\left(\frac{a^{j}}{j!}\right)}^{2}\\ & +\frac{\varphi_{2}^{2}}{1-\rho^{2}}\left[\frac{1-\rho^{2(k+1)}}{{\left(\varphi_{1}+\varphi_{2}\frac{1-\rho^{k+1}}{1-\rho }\right)}^{2}}-\frac{1-\rho^{2(k+2)}}{{\left(\varphi_{1}+\varphi_{2}\frac{1-\rho^{k+2}}{1-\rho }\right)}^{2}}\right], \end{array}$$

*where the constraint satisfies $|D_{I}(P_{k+1}\left|\right|P_{k})|\leq \lambda ∥P_{k+1}-P_{k}{∥}_{1}$ (λ>0 is a constant), and $\varphi_{1}$ and $\varphi_{2}$ are mentioned in Equations (58a) and (58b).*

Likewise, it is readily seen that the MITM between the queue state stationary probability distributions $P_{\infty }=\{p_{\infty ,0},p_{\infty ,1},\ldots ,$$p_{\infty ,\infty }\}$ and $P_{k}=\left\{p_{k,0},p_{k,1},\ldots ,p_{k,s+k},0,0,\ldots ,0\right\}$ is given by

(60) $$\begin{array}{c} D_{I}(P_{\infty }\left|\right|P_{k})\\ =\left(\frac{1}{{\left(\varphi_{1}+\varphi_{2}\frac{1-\rho^{k+1}}{1-\rho }\right)}^{2}}-\frac{1}{{\left(\varphi_{1}+\frac{\varphi_{2}}{1-\rho }\right)}^{2}}\right)\sum_{j=0}^{s-1}{\left(\frac{a^{j}}{j!}\right)}^{2}+\frac{\varphi_{2}^{2}}{1-\rho^{2}}\left[\frac{1-\rho^{2(k+1)}}{{\left(\varphi_{1}+\varphi_{2}\frac{1-\rho^{k+1}}{1-\rho }\right)}^{2}}-\frac{1}{{\left(\varphi_{1}+\frac{\varphi_{2}}{1-\rho }\right)}^{2}}\right]. \end{array}$$

In this case, the buffer selection problem in MEC can be formulated as

(61) $$\begin{array}{c} k_{I}^{*}=\min_{k}\left\{k;\right|D_{I}(P_{\infty }\left|\right|P_{k})|\leq \delta \}, \end{array}$$

where δ>0 is the threshold of variation in former difference.

In particular, if there is only one server, the corresponding queue model is M/M/1/k, it is not difficult to obtain

(62) $$\begin{array}{cc} D_{I(s=1)}(P_{\infty }\left|\right|P_{k}) & =\frac{2a}{1+a}-\frac{2a(1-a^{k+1})}{\left(1+a\right)\left(1-a^{k+2}\right)}, \end{array}$$

where $D_{I(s=1)}(P_{\infty }\left|\right|P_{k})$ denotes the MITM with the number of server s=1. The corresponding optimal buffer size is given by

(63) $$\begin{array}{c} k_{I(s=1)}^{*}=\frac{\ln\frac{\delta \left(1+a\right)}{a(2+\delta -(2-\delta )a)}}{\ln a}-1. \end{array}$$

It is apparent that δ plays an important role in selecting the caching size when using finite size caching to imitate the infinite caching working mode.

### 4.2. KL Divergence in the Queue Model

As a common information measure, KL divergence is also considered to be applied to measuring the information distinction between the queue state stationary probability distributions with different buffer sizes in the queue models. In particular, for the M/M/s model, we have the following proposition.

**Proposition** **6.** *In the M/M/s model, the KL divergence between the queue state distribution $P_{k+1}=\{p_{k+1,0},p_{k+1,1},\ldots ,$$p_{k+1,s+k+1},0,\ldots ,0\}$ and $P_{k}=\{p_{k,0},p_{k,1},\ldots ,p_{k,s+k},0,0,\ldots ,$0} with buffer size k+1 and k, is derived as*

(64) $$\begin{array}{cc} D(P_{k}||P_{k+1}) & =\sum_{j}p_{k,j}\log\frac{1}{p_{k+1,j}}-\sum_{j}p_{k,j}\log\frac{1}{p_{k,j}}\\ & =\sum_{j=0}^{s-1}\frac{a^{j}}{j!}p_{k,0}\log\frac{p_{k,0}}{p_{k+1,0}}+\sum_{j=s}^{s+k}\frac{a^{s}}{s!}\rho^{j-s}p_{k,0}\log\frac{p_{k,0}}{p_{k+1,0}}\\ & =\log\left\{1+\frac{\varphi_{2}\frac{\left(1-\rho \right)\rho^{k+1}}{1-\rho^{k+1}}}{\frac{\varphi_{1}\left(1-\rho \right)}{1-\rho^{k+1}}+\varphi_{2}}\right\}, \end{array}$$

*where the parameters $p_{k,j}$, $p_{k+1,j}$, $\varphi_{1}$ and $\varphi_{2}$ are the same as them in Proposition 5.*

Furthermore, it is not difficult to have the KL divergence between the distribution $P_{\infty }=\{p_{\infty ,0},p_{\infty ,1},\ldots ,$$p_{\infty ,\infty }\}$ and $P_{k}=\left\{p_{k,0},p_{k,1},\ldots ,p_{k,s+k},0,0,\ldots ,0\right\}$ with buffer size *∞* and *k*, which is obtained as

(65) $$\begin{array}{cc} D(P_{k}||P_{\infty }) & =\sum_{j}p_{k,j}\log\frac{1}{p_{\infty ,j}}-\sum_{j}p_{k,j}\log\frac{1}{p_{k,j}}\\ & =\log\frac{p_{k,0}}{p_{\infty ,0}}\\ & =\log\frac{\varphi_{1}+\varphi_{2}\frac{1}{1-\rho }}{\varphi_{1}+\varphi_{2}\frac{1-\rho^{k+1}}{1-\rho }}. \end{array}$$

Similar to Equation (61), it is rational for us to use KL divergence as measurement to select the buffer size. The corresponding optimal buffer size can be described as

(66) $$\begin{array}{c} K_{KL}^{*}=\min_{k}\{k;|D(P_{k}\left|\right|P_{\infty })|\leq \delta \}, \end{array}$$

where δ>0 is a small enough parameter and it can adjust the information transfer gap between the queue state stationary distributions $P_{\infty }$ and $P_{k}$ which are with buffer size *∞* and *k* respectively. Then, we have

(67) $$\begin{array}{c} k_{KL}^{*}=\frac{\ln\left(1-\frac{\left(1-\rho \right)\varphi_{1}+\varphi_{2}-2^{\delta }\varphi_{1}\left(1-\rho \right)}{2^{\delta }\varphi_{2}}\right)}{\ln\rho }-1, \end{array}$$

where *k* is the buffer size or queue length, $\varphi_{1}$ and $\varphi_{2}$ are mentioned in Equations (58a) and (58b). What is more, as for the M/M/1/k model, the optimal buffer size is simplified as follow

(68) $$\begin{array}{c} k_{KL}^{*}=\frac{\ln(1-\frac{1}{2^{\delta }})}{\ln a}-2. \end{array}$$

Therefore, by using the information measures such as MITM and KL divergence, it may provide an effective method to select the caching size, which can exploit the resources of MEC more reasonably.

### 4.3. Numerical Validation

To validate our derived results in theory, we take some event simulation experiments of the queue model by use of Matlab. By setting the arrival rate $\tilde{\lambda }$ and service rate $\tilde{\mu }$ of queue model, the process of arrival and departure for each event is simulated during a fixed period. We will elaborate on specific parameters of the queue model in the following context. In the figures of results, the legends $D_{I}$-Sim, $D_{I}$-Ana and *D*-Sim, *D*-Ana are used to denote the simulation results and the analytical results for MITM and KL divergence, respectively.

#### 4.3.1. Effect of the Traffic Intensity

**Figure 5:**
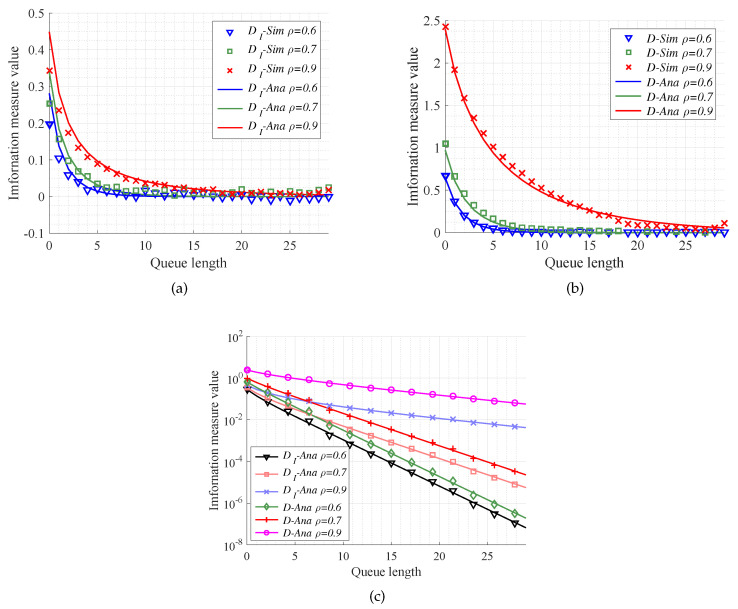
The performance of different information measures versus queue length. The queue models are with the same number of server s=1 and different traffic intensity ρ (ρ = 0.6, 0.7, 0.9). (**a**) The performance of message importance transfer measure (MITM) mentioned in Equation (60) in the case of traffic intensity ρ=0.6,0.7,0.9; (**b**) The performance of KL divergence mentioned in Equation (65) in the case of traffic intensity ρ=0.6,0.7,0.9; (**c**) The analysis results of different information measures between the queue length *k* and *∞*.

We now exploit M/M/s/k model to investigate performance of the MITM and KL divergence in the case of different traffic intensity. In the simulations, the queue length, namely the buffer size, is set to change from 0 to 30, the number of servers satisfies s=1, and the traffic intensity is set as ρ=0.6,0.7,0.9. Then, we can compare the simulation results with the theoretical ones for the MITM and KL divergence. From Figure 5, it is seen that the analytical results mentioned in Equations (59) and (60) can validate the simulation results. In particular, Figure 5a,b shows that analytical results of MITM and KL divergence can absolutely fit the simulation experiments in the M/M/s/k models with different traffic intensity. What is more, from Figure 5c, we can see that in the same queue model the convergence for MITM is faster than that for KL divergence. That is, the MITM offers a reasonably lower bound for queue length selection with respect to MEC. Besides, the less traffic intensity we have, the more caching size resources can be saved.

#### 4.3.2. Effect of the Number of Servers

With regard to effects of number of servers on the MITM and KL divergence, we do the simulation experiments with M/M/s model by setting the number of servers as s=1,3,5. What is more, we set the queue length as k=0,1,2,…30, and the traffic intensity always as ρ=0.9. Then, we gain the comparison between the simulation results and the theoretical ones.

**Figure 6:**
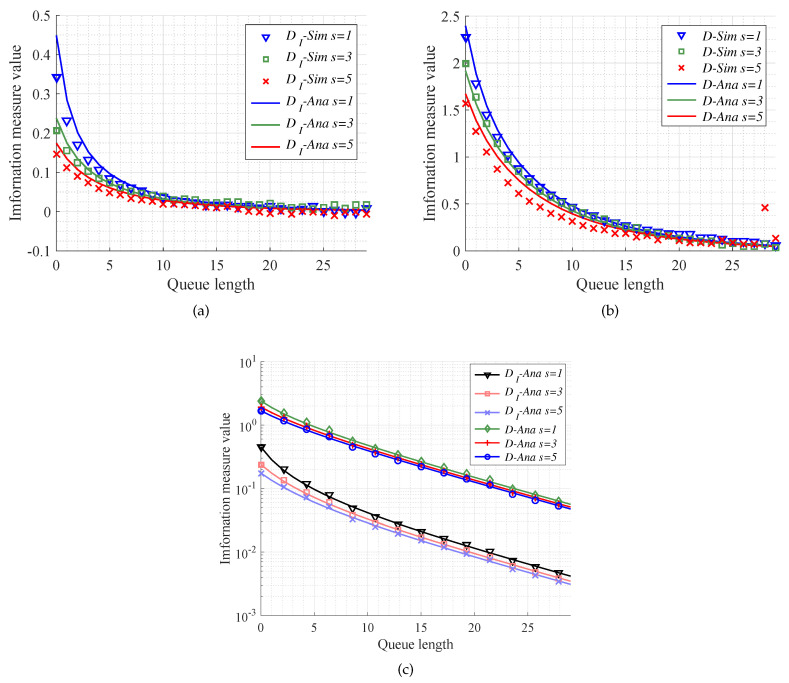
The performance of different information measures versus queue length. The queue models are with the same traffic intensity ρ=0.9 and different number of servers (*s* = 1, 3, 5). (**a**) The performance of MITM mentioned in Equation (60) in the case of the number of servers s=1,3,5; (**b**) The performance of KL divergence mentioned in Equation (65) in the case of the number of servers s=1,3,5; (**c**) The analysis results of different information measures between the queue length *k* and *∞*.

Figure 6a,b show that it is almost available for analytical results to fit simulation results. From Figure 6c, similar to Figure 5c, we can also use the MITM to gain a lower bound for queue length selection than KL divergence. Moreover, keeping other conditions the same, a larger number of servers can make MITM and KL divergence converge faster. In other words, there is a trade-off between the number of servers and caching size.

#### 4.3.3. Performance Results for Different Arrival Events Distributions

Now we discuss the performance results in the cases of different distributions of events’ arrivals which is listed in Table 1. It is apparent that average interarrival time is maintained as the same, namely $1/{\tilde{\lambda }}_{0}$. As well, we make sure that the number of server and traffic intensity are s=1 and ρ=0.9 in all cases, respectively. Then, we make simulations in the three cases to compare the testing results with the analytical results.

**Figure 7:**
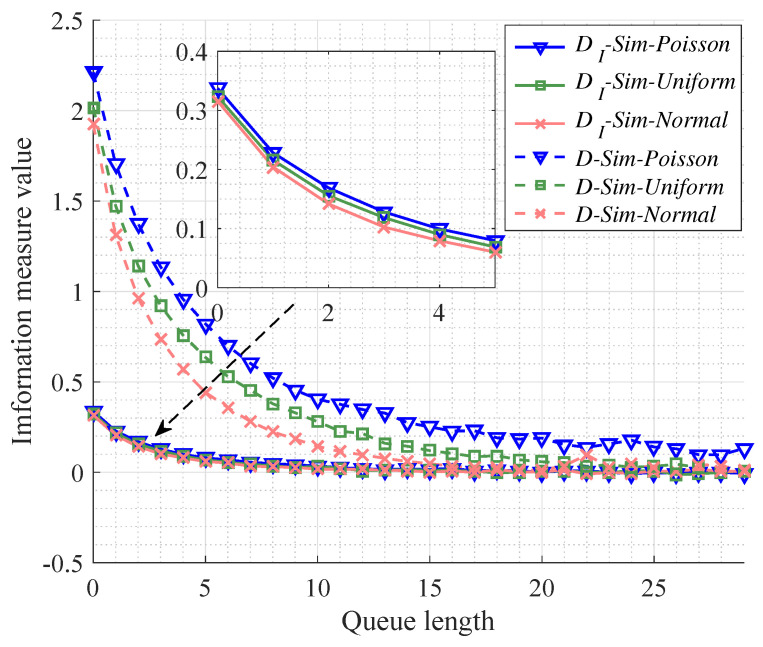
The performance of different information measures between the queue length *k* and *∞* for the queue models with the same number of server s=1, the same traffic intensity ρ=0.9, and the different arrival events’ distributions.

As for Figure 7, it is illustrated that the convergence of MITM is faster than that of KL divergence, which indicates that MITM may provide a reasonable lower bound to select the caching size for MEC. In addition, we can see that the Poisson distribution (namely, events’ arrivals follow exponential distribution) corresponds to the worst case for the arrival process among the three discussed cases with respect to the convergence of both MITM and KL divergence.

## 5. Conclusions

In this paper, the information transfer problem was investigated from the perspective of information theory and big data analytics. An information measure, i.e., MITM, was proposed to characterize the information distance between two distributions, similar to KL divergence and Renyi divergence. Actually, the information measure plays a vital role in focusing on the message importance hidden in small probability events of big data. Therefore, it is applicable for the information measure to characterize information transfer process in big data. We have investigated the variation of message importance in the information transfer process by using MITM. Furthermore, we proposed the message importance transfer capacity based on the MITM so that an upper bound can be presented for the information transfer process with disturbance. In addition, we applied the information transfer measure to select the caching size in MEC. As the next step of research, we shall carry out real data experiments to test some of the most complicated cases of MEC and make use of the information transfer measures to investigate some related algorithms as well as to discuss the effect of window length on the whole system performance in big data analytics.

## Figures and Tables

**Table 1:** The interarrival time distributions of events’ arrivals.

Type of Distribution	Exponential Distribution	Uniform Distribution	Normal Distribution
P(X)	$X∼E({\tilde{\lambda }}_{0})$	$X∼U(0,2/{\tilde{\lambda }}_{0})$	$X∼N(\frac{1}{{\tilde{\lambda }}_{0}},\frac{1}{{{\tilde{\lambda }}_{0}}^{2}})$
